# Traumatic retrosternal hematoma leading to extra-pericardial cardiac tamponade—Case report

**DOI:** 10.1016/j.ijscr.2019.06.055

**Published:** 2019-06-27

**Authors:** Rami Issam Arabi, Abdullah Aljudaibi, Abdullah Abdulaziz Althumali, Badr Saeed Rajb, Rawad Daniel Arja

**Affiliations:** a Ibn Sina National College for Medical Studies, Jeddah, Saudi Arabia; b King Abdulaziz Hospital, General surgery Jeddah, Saudi Arabia; c King Abdulaziz Hospital, Vascular surgery Jeddah, Saudi Arabia

**Keywords:** Trauma, Tamponade, Hematoma

## Abstract

**Introduction:**

Cardiac tamponade typically results from fluid or gas collection in the pericardial space leading to impairment in the cardiac function.

**Presentation of case:**

A 34 years old male patient presented to the ER after a fall from height. X-rays were done which showed no hemothorax or pneumothorax and multiple stable pelvic fractures. Computed tomography (CT) scan for trauma was done after stabilizing the patient and showed sternal fracture with a huge retrosternal hematoma. The patient was intubated immediately, and an Echocardiogram was ordered along with preparation to go to the operation room urgently due to high suspicion of a rare case of cardiac tamponade. but the patient had cardiac arrest and couldn't be revived despite the CPR effort.

**Discussion:**

This is a very unusual presentation of cardiac tamponade, mediastinal hematoma leading to extra pericardial tamponade. CT scan is an effective method to diagnose extra pericardial tamponade. In addition, to the high clinical suspicion is required. The sole treatment of mediastinal hematoma is an urgent evacuation.

**Conclusion:**

A careful assessment with high clinical suspicion along with CT Scan is the best way to diagnose extra pericardial tamponade cause by sternal fracture.

## Highlights

• Mediastinal hematoma caused by sternal fracture leading to cardiac tamponade it’s a rare presentation.
• CT Scan it’s an effective method to diagnose mediastinal hematoma.
• High clinical suspicion of extra pericardial tamponade is required when sternal fracture associated with mediastinal hematoma.
• The best management for mediastinal hematoma its urgent evocation of the hematoma to decrease the pressure on the heart.

## 1 Introduction

Cardiac tamponade is an acute life-threatening condition caused by pericardial fluid or gas collection in the pericardial sac that leads to elevation of intrapericardial pressure. in which the elevated intra-pericardial pressure will lead to cardiac compression affecting the natural pumping activity of the heart [1]. Cardiac tamponade after trauma is a well-known entity that has been reported in literature [2]. It's unreliable to diagnose cardiac tamponade depending on the triad of elevated jugular vein pressure, muffled heart sound and hypotension in the emergency situations [3] as the primary investigation method to diagnose pericardial effusion or tamponade is Doppler echocardiography [1].

Presented here a case followed the SCARE criteria [4] of a 34 years old male patient who fell from the 4th floor and was hemodynamically compromised due to the formation of retrosternal hematoma compressing the heart associated with a sternal fracture that leads to extra-pericardial tamponade which is an unusual presentation after blunt chest trauma.

## 2 Case report

**Fig. 1:**
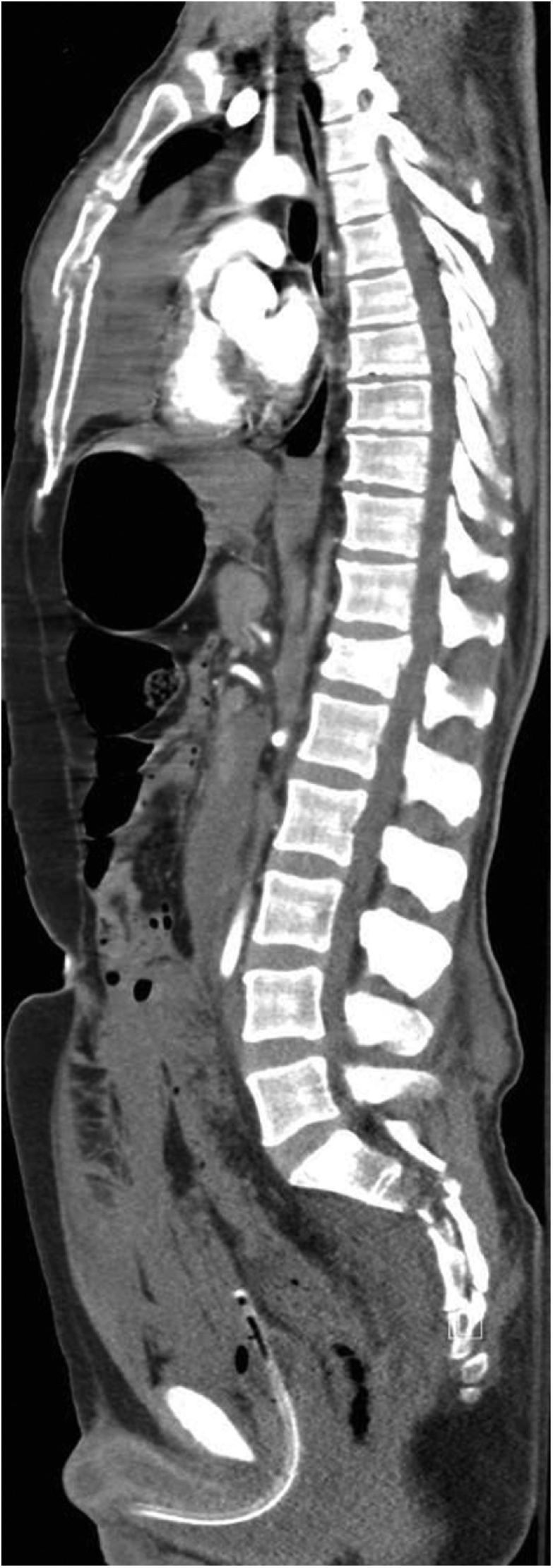
CT scan demonstrates a sternal fracture with Retrosternal hematoma.

A 34 years old male patient presented to ER after a fall from height (4th floor) impacting on his limbs and chest. Upon primary survey his airway was patent with equal bilateral air entry. Breathing was spontaneously with bilateral chest rise, oxygen saturation was 98% on room air, respiratory rate (RR) 28 breaths/min, Blood pressure (hypotension 77/48 mmHg) pulse rate 154 beats/min with raised jugular venous pressure, muffled heart sound and pelvic was stable with multiple fracture. Electrocardiography (ECG) showed low-voltage complexes. The abdomen was soft and lax with no tenderness and GCS was 15/15. Two large wide bore cannula were inserted, and the patient resuscitated with ringer lactate and one unit of packed red blood cells which helped to stabilize the patient. X-rays were done which showed no hemothorax or pneumothorax and multiple pelvic fractures. Focused assessment with sonography in trauma (FAST) and Extended FAST (eFAST) was done which showed no hemoperitoneum, no signs of pleural effusion, no apparent solid organ injury and the cardiac window was free with no fluid collection in the pericardium. Arterial blood gas (ABG) was within normal range pH: 7.4, PaCO2: 39 mmHg, PaO2: 94 mmHg. Foleys catheter was inserted and initially brought 150 ccs of clear urine. Log roll and per rectal examination (PR) were unremarkable. Initial labs tests showed hemoglobin 14.54 g/dL, white blood cells count 23 × 109 per liter (L), platelet count 299 × 103/microliter, Creatinine: 190 μmol/L. Computed tomography (CT) scan with intravenous (IV) contrast for trauma was done after stabilizing the patient, and showed sternal fracture with huge retrosternal hematoma (Fig. 1), no solid organ injury, no hemoperitoneum, no pneumothorax or hemothorax, multiple pelvic fractures, right 1 st rib fracture, right perinephric hematoma, compression fracture of D12, right transverse process fracture of L5 and multiple limbs fractured. After 1 h the patient started to deteriorate rapidly and became hypotensive 70/40 mmHg, pulse rate 130 beats/min, RR: 27 and oxygen saturation was still 98% with a patent airway and bilateral equal air entry. Resuscitation was started the massive transfusion protocol with two units of packed RBC, two units of fresh frozen plasma (FFP) and two units of platelets. The patient was intubated immediately, and an Echocardiogram was ordered along with preparation to go to the operation room urgently due to high suspicion of a rare case of cardiac tamponade most likely related to the earlier CT finding of retrosternal hematoma but the patient had cardiac arrest and couldn't be revived despite the CPR effort.

## 3 Discussion

Cardiac tamponade is a well-known condition associated with fluid or gas collection in the pericardial space. Mediastinal hematoma leading to cardiac tamponade had been described in various clinical conditions including cardiac tamponade post-open heart surgery [5], following cardiac and mediastinal penetration [6], and anterior mediastinal hematoma caused by blunt trauma [7,8].

The mortality rate from acute traumatic cardiac rupture is high. Yet, a high clinical suspicion with the presentation of cardiac tamponade may improve the prognosis [9]. However, cardiac tamponade can present from unusual mechanisms such as extra pericardial hematoma that compress the heart and present as tamponade features which are beck triad (hypotension, rise JVP and muffled heart sound) despite normal cardiac window in FAST. And normal x-ray finding. Form our case the best diagnostic feature is CT scan which showed us clearly the hematoma beneath the sternum. In addition, a high clinical suspicion of extra pericardial tamponade is required to diagnose such a case.

The time of the deterioration of the vital sign vary from patient to patient for example case reported in Italy showed a hemodynamically stable patient presented with an anterior mediastinal hematoma on CT scan with normal standard radiological finding the patient was stable for 3 h until deterioration started [10]. However, in our case after one hour of resuscitation patient start to deteriorate immediately due to the extra-pericardial compression

The intimidation for mediastinal hematoma is due to compression of vascular structure such as heart, blood vessels, and source of blood loss. Damage to the internal mammary artery, intercostal arteries, mediastinal vessels and fracture of the ribs and sternum is the main cause of mediastinal hematoma [10,11].

The risk of heart and aortic injuries increase if its associated with 1 st rib, 2nd rib, scapular or sternal fracture and the chest hitting the steering wheel during road traffic accidents is considered one of the most common causes of sternal fracture. Yet, sternal fracture causing tamponade is rarely reported, only Coleman et al. and Kao, C.L. reported two cases of sternal fracture resulting in mediastinal tamponade [12].

A CT scan is considered as an effective diagnostic test for mediastinal hematoma [11]. High clinical suspicion of extra-pericardial tamponade is required when the sternal fracture is associated with mediastinal hematoma, although, cardiac tamponade due to mediastinal hematoma tends to be more insidious when compared to pericardial tamponade mainly due to the large space of mediastinum. therefore, the only treatment for a mediastinal hematoma is urgent surgery to evacuate the hematoma [12].

## 4 Conclusion

We present a case of extra pericardial cardiac tamponade caused by a sternal fraction and subsequent sternal hematoma. The correlation between the physical finding and the standard radiological finding x-ray and Focused assessment with sonography in trauma (FAST) is not always evident. CT scan is the most effective method to diagnose extra pericardial cardiac tamponade. Clinical suspicion is mandatory to diagnose such a case.

## Conflicts of interest

No conflict of interest between authors.

## Sources of funding

No sources of funding for our paper.

## Ethical approval

Ethical approval has been taken from the ministry of health.

## Consent

Unfortunately, the patient deceased and we couldn't find any of his relatives because the patient he is non-Saudi. However, The hospital/legal team assume full responsibility for this paper and ensure that all the patient information has been anonymized.

## Author contribution

Abdullah Aljudaibi: Co-wrote the discussion part, reviewed the manuscript critically.

Rami Arabi: wrote the introduction, co-wrote the discussion, reviewed the manuscript critically.

Abdullah Althumali MD: Final approval of the manuscript.

Badr Rajb MD: reviewed the manuscript critically.

Rawad Arja MD: reviewed the manuscript critically.

## Registration of research studies

N/A.

## Guarantor

Abdullah Ghazi aljudaibi. MD.

Rami Arabi. MD.

## Provenance and peer review

Not commissioned, externally peer-reviewed.
